# The Versatile Role of Peroxisome Proliferator-Activated Receptors in Immune-Mediated Intestinal Diseases

**DOI:** 10.3390/cells13201688

**Published:** 2024-10-12

**Authors:** Edit Posta, Istvan Fekete, Istvan Varkonyi, Eva Zold, Zsolt Barta

**Affiliations:** 1 GI Unit, Department of Infectology, Faculty of Medicine, University of Debrecen, Bartok Bela Street 2-26, 4031 Debrecen, Hungary; varkonyi.istvan@med.unideb.hu (I.V.); barta@med.unideb.hu (Z.B.); 2 Institute of Food Technology, Faculty of Agricultural and Food Sciences and Environmental Management, University of Debrecen, Böszörményi út 138, 4032 Debrecen, Hungary; feketei@agr.unideb.hu; 3 Department of Clinical Immunology, Institute of Internal Medicine, Faculty of Medicine, University of Debrecen, Móricz Zsigmond str. 22, 4032 Debrecen, Hungary; zold.eva@med.unideb.hu

**Keywords:** PPAR, Crohn’s disease, ulcerative colitis, eosinophilic gastrointestinal disorders, allergic diseases, microbiome, inflammation, celiac disease

## Abstract

Peroxisome proliferator-activated receptors (PPARs) are nuclear receptors that sense lipophilic molecules and act as transcription factors to regulate target genes. PPARs have been implicated in the regulation of innate immunity, glucose and lipid metabolism, cell proliferation, wound healing, and fibrotic processes. Some synthetic PPAR ligands are promising molecules for the treatment of inflammatory and fibrotic processes in immune-mediated intestinal diseases. Some of these are currently undergoing or have previously undergone clinical trials. Dietary PPAR ligands and changes in microbiota composition could modulate PPARs’ activation to reduce inflammatory responses in these immune-mediated diseases, based on animal models and clinical trials. This narrative review aims to summarize the role of PPARs in immune-mediated bowel diseases and their potential therapeutic use.

## 1. Introduction

Nuclear hormone receptors mainly sense lipophilic molecules—not only endogenous steroid and non-steroid hormones but also exogenous ligands, dietary components, and drugs. Peroxisome proliferator-activated receptors (PPARs) exert multiple effects on inflammatory processes, regulation of lipid and glucose homeostasis, and cell proliferation by regulating target genes as transcription factors. PPARs (PPARα, -γ, and -δ/β) are lipid sensors. Historically, the first receptor studied was PPARα in 1990, and its activation led to the proliferation of peroxisomes [1]. 

The expression of PPAR isoforms has a variable distribution; PPARα and its target genes are detected in the liver and small intestine, predominantly in the duodenum and jejunum [2,3]. PPARγ, a key regulator of adipogenesis, is highly expressed in adipose tissue, the duodenum, and the proximal colon [3]. PPARδ, also known as PPARβ, is expressed ubiquitously, not just in the gastrointestinal tract. Based on animal and in vitro studies, PPARδ activation leads to the production of glucagon-like peptide-1 (GLP-1) and plays a role in the regulation of fatty acid and glucose metabolism and insulin sensitivity [4,5]. 

PPARs play a role in many pathological conditions, e.g., vascular inflammation, atherosclerosis, diabetes mellitus (DM), metabolic syndrome, and inflammatory and autoimmune diseases such as inflammatory bowel disease (IBD) [6], and they are influenced by the gut microbiota and dietary patterns [7]. 

The aim of this narrative review is to summarize the role of PPARs in the pathogenesis of IBD and other inflammatory conditions of the gut, particularly in relation to the gut microbiota and diet, and to identify drugs that may modulate the pathogenesis and inflammation of these diseases.

## 2. Peroxisome Proliferator-Activated Receptors and Immune Mediated-Gut Diseases

### 2.1. Immune-Mediated Gut Diseases 

#### 2.1.1. Crohn’s Disease and Ulcerative Colitis 

IBDs are multifactorial immune-mediated diseases involving chronic inflammation of the gastrointestinal tract due to abnormal immunological responses to the gut microbiome. The pathogenesis involves genetic susceptibility, dietary components, Western lifestyle, infections, environmental factors, etc. 

Ulcerative colitis (UC) affects the colonic mucosa, most commonly the rectal mucosa, but the inflammation can also affect the sigmoid colon (proctosigmoiditis) or the whole colon (pancolitis). CD and UC can have extraintestinal manifestations (e.g., musculoskeletal, skin, eye, oral, genital) that complicate treatment. Both of these IBDs are associated with an increased risk of colorectal cancer (CRC) and increased mortality compared to the healthy population. The average age of diagnosis is between 20 and 40 years. The exact diagnosis is based on a combination of clinical, biochemical, stool, endoscopic, and histological tests [8]. 

Treatment may include 5-aminosalycilates, topical and systemic corticosteroids, immunosuppressants such as azathioprine, biological treatments, or surgery, depending on the manifestations and severity of the disease [9,10]. 

Crohn’s disease (CD) involves transmural granulomatous inflammation of any site in the gastrointestinal tract, but the most common sites are the terminal ileum and colon. In addition to transmural inflammation, CD leads to tissue injury and dysregulated wound healing of the intestinal wall. This dysregulated repair of the bowel wall causes fibrotic strictures. Fibrotic strictures occur in 50% of patients with CD but rarely in patients with UC [11]; they can also form in other immune-mediated intestinal diseases, e.g., microscopic colitis or eosinophilic gastrointestinal disorders. Due to the continuous chronic inflammation, tissue injury occurs, and this process plays a key role in the polarization of macrophages and myofibroblasts. Various factors are responsible for macrophage polarization, including cytokines and various ligands. Macrophage polarization towards M1 is induced by microbial products such as lipopolysaccharide and pro-inflammatory cytokines such as interferon-gamma (IFN-γ). M1 macrophages produce pro-inflammatory cytokines, e.g., tumor necrosis factor-alpha (TNF-α) or interleukin-12 (IL-12); their roles are in antitumor activity, inflammatory cytokine secretion, and antibacterial defense. The other type of macrophage polarization is towards M2 macrophages, which secrete various cytokines, e.g., IL-4, IL-13, transforming growth factor-β1 (TGF-β1), and platelet-derived growth factor (PDGF), which promote the proliferation and activation of myofibroblasts and play a crucial role in wound healing [12]. Activated M2 macrophages (M2a) induce myofibroblast activation and extracellular matrix (ECM) overproduction, leading to ECM accumulation. IL-13-induced TGF-β1 plays a critical role in the fibrogenic signaling process in a Smad2/3-, Smad4-, and Smad-independent manner via p38 mitogen-activated kinase and c-Jun pathways [11]. ECM stiffness is an important regulator of myofibroblast activity and ECM production. Pathological matrix stiffness is a factor in the auto-propagation of fibrotic stricture formation, independent of inflammation [13]. Not only innate immune cells and myofibroblasts but also mesenteric fat-derived adipokines (e.g., leptin) play a regulatory role in the polarization and activation of intestinal macrophages and lead to the progression of fibrosis. In addition, the extension of mesenteric fat around the intestinal wall (creeping fat) is pathognomonic for CD [14]. There is currently no biomarker in clinical use to predict the formation of strictures. It is important to predict the formation of strictures because they often develop without symptoms. In the genetic background, two *NOD2* gene variants could predispose individuals to stricture formation [15], and at the epigenetic level, several miRNAs could be associated with the formation of strictures, but in clinical use, their measurement is not recommended [11]. 

#### 2.1.2. Microscopic Colitis 

Microscopic colitis (MC) is a non-destructive IBD that has two forms: lymphocytic and collagenous colitis. Diagnosis is based on histology with non-specific symptoms. Most patients are elderly, with only 25% of patients under 45 years of age. MC is a multifactorial disease; bile acid malabsorption, pathological expression of aquaporin channels, the use of NSAIDs and PPIs, and stress play a role in the pathogenesis. MC may be associated with other autoimmune diseases: MC patients have a higher risk of celiac disease (50–70-fold), autoimmune thyroiditis (3-fold), and enteropathic arthritis [16]. Topical corticosteroids, cholestyramine, thiopurines, or biologics can be used for the treatment. There is little evidence for the use of mesalamine, prednisolone, methotrexate, and, finally, surgery [17]. 

#### 2.1.3. Celiac Disease 

Celiac disease (CeD) is an autoimmune disease triggered by wheat gliadin in genetically predisposed (i.e., HLADQ2/DQ8-positive) individuals. CeD has a wide range of manifestations, from asymptomatic disease to severe malabsorption, weight loss, chronic diarrhea, bloating, abdominal pain, and arthralgia, with a high risk of developing Hodgkin’s lymphoma and gastrointestinal cancers, e.g., small-intestinal adenocarcinoma. Diagnosis is based on clinical manifestations with positive serological markers, genetic susceptibility, and small intestine histology. Typical histological findings include villous atrophy, crypt hyperplasia, and lymphocytic infiltration of the epithelium and lamina propria. A lifelong strict gluten-free diet (GFD) is the only effective therapy, but 5% of patients are non-responders [18]. 

#### 2.1.4. Eosinophilic Gastrointestinal Disorders

Eosinophilic gastrointestinal diseases (EGIDs) are chronic inflammatory disorders characterized by the accumulation of eosinophil granulocytes at specific sites of the gastrointestinal tract. The name depends on the localization of the eosinophilic inflammation, eosinophilic esophagitis, eosinophilic gastritis, etc.

The diagnostic criteria for eosinophilic esophagitis include symptoms of esophageal dysfunction, endoscopy showing >15 eosinophils per high-powered field from the esophagus, and exclusion of other diseases (e.g., infection, IBD, systemic autoimmune diseases, achalasia, and gastroesophageal reflux disease). Eosinophilic esophagitis and other localized EGIDs have an increasing prevalence. There is a strong association between dietary components and atopic diseases. There is a typical Th2 imbalance leading to increased secretion of IL-5, IL-13, IL-4, and chemokines; these processes cause basal cell hyperplasia, eosinophilic granulocyte infiltration, and fibroblast activation. The result is collagen deposition in the lamina propria and tissue stiffness. If left untreated, this leads to gastrointestinal fibrosis and strictures. Mast cells play a role in this pathogenesis, and some patients have elevated levels of IgE in their peripheral blood. Treatment of these diseases is difficult; corticosteroids, proton-pump inhibitors, azathioprine, antihistamines, leukotriene antagonists, and elimination diets may be helpful. For esophagitis, endoscopic dilatation or stent placement could be a solution [19,20]. The IL-5 inhibitor mepolizumab failed to control the disease despite a reduction in eosinophil count. The IL-13 inhibitor cendakimab, a humanized monoclonal antibody, may be safe, with good clinical outcomes based on a phase III study. The IL-4 receptor alpha (IL-4R*α*) component inhibitor dupilumab is a fully human monoclonal antibody that is currently approved by the European Medicines Agency (EMA) and the Food and Drug Administration (FDA) for the treatment of eosinophilic esophagitis, but it can induce histological and clinical remission in other localizations, such as eosinophilic gastritis and duodenitis, based on small retrospective studies [21,22].

#### 2.1.5. Allergic Disorders of the Gastrointestinal System

These allergic diseases are a spectrum of disorders characterized by immune-mediated reactions, predominance of Th2 immunity, loss of tolerance to food antigens, and impaired barrier function (food allergy and anaphylaxis risk are associated with impaired skin barrier function and may manifest as atopic dermatitis in early childhood). 

Allergic gastrointestinal diseases are classified according to IgE-mediated, non-IgE-mediated, and mixed IgE-mediated and non-IgE-mediated mechanisms. In IgE-mediated allergic diseases, there is an increased Th2-mediated imbalance with impaired Treg functions, and this imbalance leads to an IgE-producing switch in B cells. IgE binds to FcεRI on mast cells, and food antigens crosslink these receptor–IgE complexes, leading to the activation and degranulation of mast cells. The offending antigens are peanuts, soy, fish, shellfish, milk, eggs, wheat, beef, and chicken. Interestingly, red meat allergy is also associated with tick bites. Fruit and vegetable allergy is caused by a pollen-derived epitope. Diagnosis is based on clinical symptoms, skin prick test, serum-specific IgE, and novel diagnostic tools such as allergen component-resolved diagnostic testing (CRD), microarrays, and the basophil activation test (BAT) [23]. 

IgE- and non-IgE-mediated (mixed) allergic disorders, known as eosinophilic gastrointestinal disorders, are described above. 

Non-IgE-mediated food allergies include food-protein-induced enterocolitis and proctocolitis syndromes. The offending foods are milk, soy, wheat, oats, rice, chicken, turkey, and eggs. Diagnosis is based on clinical symptoms: a good response to an allergen elimination diet and recurrence of symptoms on challenge test; diagnosis by prick test and IgE levels is not viable. In cases of rectal bleeding, fecal blood tests, colonoscopy, and sigmoidoscopy are also recommended [24,25].

### 2.2. PPARα

#### 2.2.1. PPARα’s Structure, Ligands, and Effects

The human *PPARα* gene is located in the 22q12-q13.1 region on chromosome 22; it spans 93.2 kb and encodes 468 amino acids [26]. The corresponding murine *PPARα* gene is also found on chromosome 15E2 [1] and shares 91% homology with the human gene, encoding 468 amino acids as well. The protein of human PPARα, and of human PPARs in general, contains six domains (A–F) with a canonical nuclear receptor structure. These domains play a role in regulating the transcriptional activity of several target genes by integrating intracellular signals. The exact roles of these domains are briefly described below. 

The A and B domains have ligand-dependent/independent activation functions and target gene specificity; the C domain can bind to peroxisome proliferator response elements (PPREs) that contain a conserved DNA sequence motif and interact with c-Jun, which is part of the AP1 transcription factor; the D domain is a hinge region that plays a key role in the structural integrity and flexibility of the nuclear receptor and the site of post-translational modification by protein kinase C (PKC); the E/F domains on the C-terminus of PPARα have ligand-binding specificity and can interact with retinoid X receptor (RXR) to form heterodimers, p65, and other co-regulators [27].

There are co-repressor molecules that bind to unliganded or antagonist-liganded heterodimers—e.g., nuclear hormone co-repressor (NCOR1), the silencing mediator of retinoic acid and thyroid hormone receptor (SMRT), and TNF-induced protein 3-interacting protein 1 (TNIP1)—and these repressors interact with histone deacetylases (HDACs). HDACs modify the histone to make it inaccessible to other transcription factors; this is defined as ligand-independent transrepression. Liganded PPAR/RXR heterodimers release the repressor molecules via conformational changes and binding to coactivators, e.g., PPAR-binding protein (PBP/PPARBP). More than 300 cofactors, co-regulators, and co-repressors have been identified, but in some cases, their exact role is uncertain [28]. 

There are two mechanisms in the regulation of gene expression by PPARs: transrepression and transactivation. In the case of transactivation, liganded PPAR/RXR heterodimers bind directly to PPREs in target genes. This results in the binding of coactivator complexes, changes in chromatin structure, and the facilitation of transcription [29].

Another regulatory mechanism is ligand-dependent transrepression. This is an important mechanism, as the anti-inflammatory functions of PPARs are based on these processes. PPARs antagonize the effects of other transcription factors, e.g., NF-κB; there are different forms, including the following: (1) direct interaction with other transcription factors, e.g., p65 subunit–PPAR interaction, in which these signaling proteins can bind to one another, preventing them from binding to their response elements and inhibiting gene transcription; (2) the induction of iκB expression, which leads to NF-κB inhibition; (3) competition for the limited pool of general coactivators, e.g., p300; (4) activation of PPARs leading to the inhibition of kinase activity, e.g., c-Jun N-terminal kinase or MAPK; and (5) the inhibition of signal-dependent co-repressor release leading to the inhibition of gene transcription [29]. 

In addition, there are several other pathways to regulate PPARs’ activity, such as various post-translational modifications, including phosphorylation, SUMOylation, ubiquitination, acetylation, and O-GlcNAcylation. Phosphorylation of specific serine residues of PPARs can modulate transactivation and interactions with co-repressor molecules. SUMO is a 12 kDa polypeptide that can covalently attach to lysine residues. SUMOylation of PPARα increases the recruitment of co-repressors. The ubiquitin-proteasome system can regulate the stabilization of PPARs and protein levels [30]. These regulatory processes of the PPAR-regulated gene expression are important in the modulation of metabolism, inflammation, and innate immune responses. For this reason, these pathways are potential targets in immune-mediated and autoimmune diseases, not just in the gastrointestinal system. 

PPARα (and the other PPARs) has potent anti-inflammatory properties and plays a role in the regulation of immune responses. When PPARα agonists are bound, they form an active PPARα/RXR heterodimer, which acts as a transcription factor to modulate PPREs. Endogenous ligands can include fatty acids (e.g., saturated palmitic and oleic acids), palmitoylethanolamide (PEA), lipoxygenase (LOX) products such as 8-hydroxyeicosatetraenoic acid (8-(S)-HETE), and leukotriene B4 (LTB4); these are products of arachidonic acid [31]. Arachidonic acid is released from membrane-bound phospholipids by phospholipase enzymes (phospholipase A2, C, D) and by subsequent enzymes. These enzymes include lipoxygenases, which metabolize arachidonic acid to leukotrienes and hydroxyeicosatrienoic acids (HETEs); cyclooxygenases, which metabolize it to prostanoids, prostacyclins, and thromboxanes; and cytochrome P450-associated enzymes, which metabolize it to HETEs and epoxyeicosatrienoic acids (EETs) [32]. These products can play roles in inflammation, cell migration, and the regulation of vasodilatation, and some of these effects are mediated by PPARα. 

The synthetic agonist GW7647 and the antagonist GW6471 are used experimentally. The pharmacological agonists are fibrates, which are widely used in clinical practice (e.g., fenofibrate, clofibrate, gemfibrozil, and ciprofibrate). 

The primary target genes of activated PPARα/RXR heterodimers are involved in mitochondrial and peroxisomal fatty acid β-oxidation (e.g., acyl-CoA dehydrogenases, acyl-CoA oxidases), ω-oxidation and ω-hydroxylation (cytochrome P450), and ketogenesis (3-hydroxy-3-methylglutaryl-CoA synthase) [27]. 

PPARα is expressed in CD45+ leukocytes and basophils, monocytes, macrophages, Kupffer cells, Langerhans cells, and microglial cells. In addition to its anti-inflammatory properties, PPARα exerts transcriptional activity on NF-κB, AP-1, and signal transducers and activators of transcription (STATs), as well as direct interactions with the c-Jun, p65, and Iκ pathways [33]. Because of these interactions, PPARα modulates the innate immune response at many points (Table 1). PPARα activation leads to the regulation of catabolism of inflammatory lipid mediators, e.g., clearance of LTB4, which is an endogenous PPARα ligand with pro-inflammatory properties (e.g., facilitating migration and extravasation) [34]. Activation of PPARα led to a decrease in IL-6 production through the interaction of c-Jun and p65, and it inhibited the production of TNF-α by cultured adipocytes [35]. 

In an in vitro study, PPARα activation by synthetic agonists decreased inducible nitric oxide synthase (iNOS/NOS2) expression and NO production in a dose-dependent manner by enhancing proteasome-mediated NOS protein degradation [38]. T-bet is a crucial regulator of IFN-γ expression in Th1 cells. PPARα negatively regulates T-bet expression via suppression of p38-mitogen-activated protein kinase (MAPK) phosphorylation [40,41]. Based on these studies, PPARα and its agonists play an essential role in the regulation of innate and adaptive immune responses. 

#### 2.2.2. The Role of PPARα in Immune-Mediated Gut Diseases 

The transcriptomic and proteomic expression analysis of human colon biopsies from CD and UC patients revealed downregulation of the PPAR pathway [42]. 

PPARα has an important regulatory function. In PPARα-KO mice, there is a commensal dysbiosis in the gut with upregulation of T-helper 1/17 (Th1/Th17) cells, which can be significantly reduced by antibiotics. PPARα-KO mice are highly susceptible to colitis. PPARα also regulates IL-22 production by innate lymphoid cell 3 (ILC3) in the colon. IL-22 plays a role in the regulation of the integrity of the intestinal epithelial barrier, as well as in the production of antimicrobial peptides [39]. In another study, PPARα-KO mice had increased inflammatory activity because PPARγ and -β/δ did not compensate for LTB4-mediated inflammatory pathways [34]. 

The direct PPARα agonist palmitoylethanolamide (PEA) is a cannabinoid-like bioactive lipid mediator that acts on TRPV1 and CB2-like receptors; it is naturally found in egg yolk and peanuts. PEA can be synthesized endogenously and elevated in inflamed tissues. As an exogenous supplement, it has beneficial anti-inflammatory effects via PPARα signaling. In high-fat diet (HFD)-induced obesity in mice, PEA modulated dysbiotic microbiota, regulated colonic tryptophan metabolism and serotonin (5-HT) turnover, and reduced the inflammatory response [43]. In an animal model of IBD (dinitrobenzenesulfonic acid-induced colitis in mice), an increased level of endogenous PEA was observed in the colon; in contrast, exogenous PEA ameliorated inflammation and intestinal permeability. It is also important to note that its anti-inflammatory effect is not only due to the PPARα pathway, as mentioned above. The anti-inflammatory effect of PEA was inhibited by treatment with PPARα, GPR55, and CB-2 antagonists [44]. In biopsies from ulcerative colitis patients, PEA levels were significantly (i.e., 1.8-fold) higher than in healthy control subjects; this suggests that the anti-inflammatory activity of the endogenous mediator PEA is realized in ulcerative colitis through the PPARα pathway in addition to the already mentioned receptors [45]. 

Another animal study investigated another antioxidant, the phenylpropanoid glycoside verbascoside, a plant biosynthetic molecule derived from phenylalanine, which acts as a radical scavenger. In PPARα-KO and WT mice, verbascoside ameliorated DNBS-induced colitis in a PPARα-mediated manner. This effect was attenuated in PPARα-KO mice compared to WT control mice [46]. 

Another antioxidant was investigated in TNBS-induced colitis in mice—another animal model of IBD; treatment with polyphenolic maqui extract from *Aristotelia chilensis* improved colonic inflammation and reduced NLRP3 inflammasome activation, IL-1β secretion, and NF-κB activation. PPARα may have played a role in this process, but not all of the results were significant [47]. 

Treatment with *Phaseolus vulgaris* extract increased butyrate levels, had anti-inflammatory effects and improved intestinal barrier dysfunction in HFD mice via the PPARα and -γ pathways [48]. 

The PPARα agonist fenofibrate, a lipid-lowering drug, improves colitis by reducing IL-17 and IFN-γ production by Th1 and Th17 cells in IL-10 -/- KO mice, a murine model of IBD [49]. Conversely, in another animal study, DSS-induced colitis in mice was exacerbated by fenofibrate treatment in a PPARα-dependent manner, based on the modulation of sphingolipid metabolism, upregulation of fatty acid β-oxidation, and RIPK3-dependent necrosis in the colon [50]. These studies used different animal models, and because of the conflicting results, further studies are needed to clarify the contradictory results. Despite controversial animal studies, there are now active phase II-III clinical trials of fibrates in UC patients, but results have not yet been reported [51,52]. 

Atorvastatin not only inhibits 3-hydroxy-3-methylglutaryl coenzyme A (HMG-CoA) reductase but also exerts pleiotropic effects, upregulating PPARα expression and leading to anti-inflammatory and antifibrotic effects. PPARα was downregulated in experimental DSS-induced colitis in mice, and PPARα levels in the colon were increased by atorvastatin treatment. UC patients treated with the TNF inhibitor infliximab had increased PPARα expression, whereas non-responders did not. PPARα could be a useful marker for predicting treatment outcomes. Atorvastatin may be beneficial in treating inflammatory bowel disease by affecting the PPARα pathway [53]. 

In a Swedish nationwide population-based case-control study, statin treatment was associated with a lower risk of Crohn’s disease but not the development of UC [44]. In another study, atorvastatin treatment was significantly associated with a reduced risk of colectomy in samples from the Stanford Research Repository (STARR) database (*n* = 827) and the Optum Clinformatics DataMart *(n* = 7821) [54]. 

In conclusion, PPARα not only modulates immune responses but may also play a role in regulating the composition of the gut microbiota [39]. Based on these studies, its agonists could play a potential complementary role in the treatment of IBD. More likely, PPARα agonists could be used as a potential biomarker for clinical outcomes, but further studies are still needed to confirm this. 

### 2.3. PPARγ

#### 2.3.1. PPARγ’s Structure, Ligands, and Functions

As mentioned above, PPARγ is highly expressed in the immune system, adipose tissue, duodenum, and proximal colon and is the best-studied of the three subtypes of PPAR. The human *PPARγ* gene is located on chromosome 3, at 3p24.2-p25, with nine exons [55]. 

PPARγ has two isoforms due to different promoters and alternative splicing: PPARγ1 is widely expressed throughout the body (white and brown adipose tissue, immune system, liver, and muscle), while PPARγ2 is expressed exclusively in adipose tissue and contains an additional 30 amino acids at the N-terminus compared to PPARγ1 [56]. 

The PPARγ protein has a conserved structure. The most conserved domain is a C domain with DNA-binding capacity [57]. 

There are various post-translational modifications of PPARγ. Activation by the MAPK pathway leads to the phosphorylation of serine 112 of PPARγ. This event decreases ligand-binding affinity and coactivator binding, and it increases the binding to period circadian regulator 2 (PER2) protein, leading to reduced PPARγ activity and decreased recruitment of target genes. In an in vitro study with the THP-1 macrophage cell line, TGF-β1/2 treatment decreased the expression levels of CD36, which functions as a scavenger receptor of oxidized LDL and plays a role in foam cell formation and atherosclerosis; this process was inhibited by MAP kinase inhibitors. TGF-β1/2 treatment led to subsequent phosphorylation of PPARγ by MAP kinase, resulting in decreased activity of PPARγ [58]. SUMOylation could interfere with the anti-inflammatory response of PPARγ. Acetylation of lysines 268 and 293 led to increased binding of the repressor NCoR to PPARγ [30]. Ubiquitination plays a role in the stability and proteasomal degradation of PPARγ [30]. O-GlcNAcylation is a single-sugar modification with β-*O*-linked *N*-acetylglucosamine (*O*-GlcNAc) to serine and threonine residues, which leads to reduced transcriptional activity of PPARγ and influences adipocyte differentiation [59]. In conclusion, post-translational modifications affect the transcriptional activity and functions of PPARγ.

Endogenous ligands of PPARγ can act as agonists, including 15S-hydroxy-5Z,8Z,11Z,13E-eicosatetraenoic acid (15-HETE), 15-keto-prostaglandin E2, 15-deoxy-Δ12,14-prostaglandin J2 (15d-PGJ2), components of oxidized low-density lipoprotein (oxLDL), and unsaturated fatty acids [60]. The endogenous antagonist is 1-acyl-2,3-cyclic glycerophosphate [61]. 15-Keto-PGE2 is an endogenous PPARγ ligand in the colon [62,63,64]. There are many endogenous PPARγ ligands, but their importance varies according to their intrinsic binding affinities.

Serotonin metabolites act as endogenous ligands. 5-Methoxy-indole acetate is an agonist of PPARγ, and monoamine oxidase plays a key role in serotonin metabolism; it is upregulated by the alternative activation of macrophages in an IL-4-dependent manner [64,65], while monoamine oxidase inhibition led to a loss of PPARγ activity in the human THP1 cell line [66].

Nitrolinoleic acid, a fatty acid nitration product, acts as an endogenous PPARγ agonist with the same potency as the synthetic agonists thiazolidinediones. Nitrolinoleic acid treatment resulted in PPARγ-dependent CD-36 macrophage expression, adipocyte differentiation, and glucose uptake [67].

The best-known synthetic ligands, the thiazolidinediones (TZDs), are synthetic agonists of PPARγ (e.g., rosiglitazone, pioglitazone). These molecules are used as antidiabetic drugs to improve insulin sensitivity, increase insulin-stimulated glucose uptake in adipose tissue and skeletal muscle, and increase lipid uptake and storage in adipose tissue; they prolong pancreatic β-cell function and inhibit β-cell apoptosis. Although rosiglitazone has improved diabetes mellitus, it also increased the risk of myocardial infarction and death [68]. In contrast, pioglitazone improved cardiovascular outcomes by 16% in the PROactive study [69]. The difference in cardiovascular risk is based on the effect of lipid fraction. Rosiglitazone significantly decreased HDL, total cholesterol, and LDL levels, while pioglitazone significantly increased HDL levels but decreased triglycerides without effect on LDL levels [62,70].

Rosiglitazone modulates innate immune functions. There have been some in vitro studies based on this feature. The NF-κB pathway was suppressed in lipopolysaccharide (LPS)-treated macrophages in vitro by rosiglitazone treatment, but this effect was not observed when the PPARγ gene was silenced [71]. Rosiglitazone decreased the expression of cyclooxygenase-1 and -2 enzymes in a hyperglycemic cell model [72]. Pioglitazone treatment significantly decreased Toll-like receptor (TLR)2 and -4 expression, as well as TLR2- and -4-ligand-induced NF-κB activation and IL-1β, IL-1, and TNF-α production, in isolated human monocytes compared to controls [58]. Pioglitazone modulated dendritic cell activation, decreased DC-SIGN expression, and suppressed the NF-κB and MAPK pathways [73,74].

TZDs play a role in modulating the relationship between immune responses and infectious agents. Rosiglitazone treatment during influenza infection and secondary bacterial infections is associated with increased mortality due to suppression of inflammation and bacterial clearance [75].

Intestinal ischemia, a severe and life-threatening condition with high mortality, is a complex pathological process; it is usually caused by mesenteric artery thrombosis, hypotension, shock, or necrotizing enterocolitis, leading to intestinal damage that progresses after reperfusion. This process, while only partially understood, involves a myriad of factors, including significant roles of oxidative stress, inflammation, TLR4, and intestinal microbiota composition [76,77].

In a murine model of ischemia–reperfusion-induced intestinal injury, significantly increased macroscopic and microscopic damage and increased MPO activity was detected in PPARγ-deficient heterozygous (+/−) mice. Rosiglitazone treatment improved colitis, decreased MPO activity, and decreased the expression of TNF-α and ICAM-1 at the mRNA level. Rosiglitazone played a protective role against colitis in this animal model. This investigation was the first evidence of the in vivo anti-inflammatory effect of PPARγ in ischemia–reperfusion injury in mice [78].

PPARγ agonists promoted the alternative activation state of macrophages (M2) and suppressed the immunoreactive state (M1). Alternative activation is an immunotolerant form to support tissue repair and angiogenesis with the typical markers CD36, IL-13, Arg1, Ym1, Fizz1, CD206, IL-4, and IL-10 [79]. PPARγ modulated the long-chain fatty acid (i.e., oxidized LDL) scavenger receptor, along with CD36 expression and function [80]. As mentioned above, this macrophage state plays an important role in the pathogenesis of pathological wound healing and fibrotic processes.

PPARγ has antifibrotic effects, and TZDs modulate the process of fibrosis. Myeloid-cell-specific PPARγ deficiency in mice led to increased severity of pulmonary fibrosis and increased lung collagen deposition after influenza A infection [81]. In another study, the macrophage-specific PPARγ-KO-/- mice showed delayed wound healing, reduced granulation tissue size and collagen deposition, reduced density of new blood vessels, increased TNF-α production, and reduced phagocytosis of apoptotic cells [82].

Another proof of the antifibrotic effect of TZDs is that hepatic stellate cells play an important role in the pathogenesis of liver fibrosis. In vitro, treatment of these cells with 5-deoxy-Δ^12,14^-prostaglandin J_2_ or with troglitazone inhibited cell proliferation and platelet-derived growth factor-induced migration in a dose-dependent manner [83]. Another TZD, pioglitazone, significantly reduced steatosis, fibrosis, and inflammation in mice with HFD-induced non-alcoholic fatty liver disease (NAFLD) [84].

In another clinical trial, pioglitazone improved histopathology and laboratory parameters (e.g., transaminases, lipid parameters, HOMA-IR) in diabetic and non-diabetic NAFLD patients (*n =* 614) [85]. The gut microbiota may play a role in this process. Pioglitazone increases linoleic acid and its metabolite 10-hydroxy-cis-12-octadecenoic acid (HYA), which is derived from the *Lactobacillus* fraction in the gut [86].

Rosiglitazone treatment inhibited paraquat-induced pulmonary fibrosis in rats in a PPARγ-dependent manner, and the GW9662 antagonist blocked this antifibrotic effect [87].

Systemic sclerosis is an autoimmune disorder associated with progressive fibrosis of the skin and multiple organs. Notably, dermal fibroblasts from systemic sclerosis patients with the diffuse cutaneous form had significantly reduced PPARγ expression compared to healthy controls, highlighting a key difference in the disease. In addition, expression of α-SMA, type I collagen, and CTGF protein was significantly increased after rosiglitazone treatment in vitro, suggesting that PPARγ modulates fibrogenesis and may have a therapeutic target in fibrosis [88]. Conversely, rosiglitazone treatment suppressed TGF-β1-induced myofibroblast activation, particularly via the p38 MAPK pathway, but this antifibrotic effect was not affected by the PPARγ antagonist GW9662; this effect was not PPARγ-dependent [89].

Based on these studies, PPARγ not only influences insulin sensitivity but also regulates innate immunity, macrophage polarization, phagocytosis, wound healing, and the modulation of tissue repair and fibrosis.

#### 2.3.2. PPARγ and Immune-Mediated Gut Diseases

##### PPARγ and IBD

PPARγ plays a role not only in the regulation of immunity, macrophage polarization, and tissue repair but also in the pathogenesis of IBD. DSS-induced colitis is a widely used animal model for the investigation of IBD. In a murine model, DSS-induced colitis was exacerbated by PPARγ-deficient macrophages—not only in the disease activity index but also in histological samples. Additionally, the experimental animal model with PPARγ-deficient macrophages showed significantly increased colonic IFN-γ expression, as well as TLR4, Ly6c, and CD40 expression in macrophages and CD8+ T-cell infiltration in the lamina propria [90].

In a different murine study, PPARγ-deficient T cells in mice with DSS-induced experimental colitis exacerbated disease onset and weight loss. Histopathological samples from these PPARγ-deficient mice showed significantly more epithelial erosions, leukocyte infiltration, and intestinal wall thickening. In mesenteric lymph node samples, flow cytometry showed more CD8+ T cells and fewer Treg cells in the blood. On mRNA microarray, IL-6, IL-1β, and SOCS3 were upregulated. On gene set enrichment analysis (KEGG database), genes of ribosomal pathways and Szentgyörgyi-Krebs-cycle were downregulated, while genes of NF-κB-dependent apoptosis were upregulated [91]. These experiments are a good example of the role of PPARγ in the regulation of the immune response and its anti-inflammatory effects.

In another animal study, researchers investigated the role of PPARγ in intestinal epithelial cells (IECs) in DSS-induced colitis in mice; they found that intestinal epithelial lesions became more severe in PPARγ-deficient mice with a mixed FVB/C57BL/6 genetic background. However, no changes in disease activity index or weight loss were observed. On the other hand, all three parameters worsened in mice with a C57BL/6 background. Microarray analysis showed decreased IL-10 secretion by CD4+ T cells in mesenteric lymph node samples. These observations suggest that PPARγ plays an immunoregulatory role in IECs and influences the inflammatory response and severity of colitis [92].

In a different animal study, treatment with pioglitazone (another PPARγ agonist) improved DSS-induced colitis, disease activity, and histology and decreased TNF-α secretion in mice in an annexin-A1-dependent manner. Annexin A1 mediates the anti-inflammatory effect of glucocorticoids, and treatment with the PPARγ agonist rosiglitazone upregulated annexin A1 expression [93].

There is some proof that the expression of PPARγ is decreased in IBD, exclusively in UC. A translational research study investigated the role of TLRs and TLR-regulatory protein; the mRNA expression of the pattern recognition receptor for bacterial lipopolysaccharide (LPS), TLR4, was not significantly increased in CD, but the difference was significant in active UC patients compared to healthy controls in frozen biopsy samples from the colon. Biopsies were taken from UC patients (*n* = 22), CD patients (*n* = 19), and healthy controls (*n* = 12). TLR4 is expressed in intestinal immune cells and intestinal epithelial cells. No significant differences in the expression of other TLRs were observed by Q-RT-PCR. PPARγ and the other TLR4-regulatory protein Tollip mRNA levels were significantly decreased in both UC and CD according to Q-RT-PCR, and its protein levels in active UC and CD were significantly decreased according to immunofluorescence [94].

In a small clinical cohort, 66 IBD biopsy samples were analyzed, consisting of 38 UC and 28 Crohn’s disease (CD) tissue samples, along with 30 healthy colorectal mucosa samples, and PPARγ expression was decreased in samples of active UC, while PPARγ was significantly related to the activity of UC but not the severity of disease or location, and not in CD. In a murine AOM/DSS-induced colitis model with +/-5-ASA treatment, PPARγ expression was significantly downregulated in the case of AOM/DSS-induced colitis without 5-ASA treatment compared to healthy controls. In this animal model, 5-ASA significantly reduced clinical disease activity and improved PPARγ expression, attenuating inflammation and antineoplastic effects [95].

A genomic study was conducted on the association of polymorphisms of PPARγ with IBD. In a Danish cohort of IBD patients with homogeneous clinical severity (CD: *n* = 624, UC: *n* = 411; healthy controls: *n* = 795), *PPARγ* gene PPARG C.G (rs1801282) polymorphism (Pro12Ala mutation) was associated with UC, but not with CD [96]. In a previous smaller Japanese study, PPARG Pro12Ala mutation was associated with higher prevalence in UC but not CD or healthy controls. In UC patients, this mutation led to decreased PPARγ gene expression in colonic mucosa and was associated with increased MyD88 TLR-4, 5, 9, p65, and TNFα in mRNA levels [97]. This is a known mutation that could decrease the transactivation potential of PPARγ and reduce adipogenesis [98].

A multicenter RCT observed the effects of rosiglitazone on the course of UC and showed the downregulation of PPARγ in inflamed lesions in UC. Patients with active, mild–moderate ulcerative colitis (*n* = 105) were treated with 4 mg of rosiglitazone (*n* = 52) and a placebo twice per day for 12 weeks between 6 September 2002 and 11 January 2006. After 12 weeks of treatment, 44% of the rosiglitazone group and 23% of the placebo group achieved the primary outcome of clinical response (Mayo score reduction of 2 points or more) (*p* = 0.04), but smoking and age were confounders by logistic regression. The correlation between rosiglitazone treatment and clinical response was stronger after adjustment for these factors. Clinical remission (Mayo score ≤ 2) was achieved in nine patients (17%) treated with rosiglitazone and one patient (2%) treated with placebo (*p* = 0.01). Based on this RCT, rosiglitazone may be an effective treatment for mild–moderate ulcerative colitis [99]. In another study of rosiglitazone in UC, PPARγ mRNA expression was reduced in the inflamed mucosa of UC patients. Fourteen patients with UC were randomized to treatment with 4 mg of rosiglitazone enema or mesalamine once per day for 14 days. The Mayo score was significantly reduced, and the anti-inflammatory effect was similar to that of topical mesalamine [100].

The role of another PPARγ agonist—pioglitazone, used as an antidiabetic drug in type 2 diabetes—in the risk of IBD was investigated in *n* = 12,763 ever users and *n* = 12,763 never users in Taiwan. There was no association between the risk of IBD and the use of metformin. However, the recommended dose of pioglitazone in Taiwan is no more than 30 mg, which was a limiting factor in this study (in Europe, the maximum dose is 45 mg daily) [101]. The investigation of the metformin joint effect was important because, according to another study, previous or combined metformin treatment reduced the risk of IBD [102].

Another study from Taiwan investigated the impact of rosiglitazone on the risk of inflammatory bowel disease (IBD) in 6226 people with type 2 diabetes who had taken rosiglitazone and 6226 people who had never taken it. The study found no connections between rosiglitazone treatment, major risk factors, or metformin use for IBD [103]. These two studies indicate that taking PPARγ agonists in another indication did not affect the risk of developing IBD, but there were limiting factors.

As previously discussed, PPARγ plays a pivotal role in the regulation of fibrosis and wound healing. Intestinal fibrosis, the formation of fibrotic strictures, is a common and serious complication of both CD and UC. Based on scientific reports, PPARγ plays a role in regulating the TGF-β pathway in a Smad-dependent and -independent manner. These pathways play a crucial role in the process of fibrosis.

In an in vitro study on human primary intestinal myofibroblasts, treatment with rosiglitazone and troglitazone inhibited TGF-β-induced α-SMA expression, collagen-1A1, and fibronectin at the mRNA and protein levels. TGF-β induced Akt and Smad2 phosphorylation. Rosiglitazone and troglitazone could inhibit this process. This antifibrotic effect was PPARγ-independent, and the PPARγ antagonist GW9662 did not reverse it [104].

5-ASA is a PPARγ agonist but has no antifibrotic properties. A 5-ASA analog with potent PPARγ agonism, GED-0507-34-Levo (GED), has 100-150 times greater anti-inflammatory activity than 5-ASA. In DSS-induced colonic fibrosis, GED inhibited macroscopic and microscopic intestinal lesions; it decreased the expression of Acta2, COL1a1, and Fn1 at the mRNA level and α-SMA and collagen I-II at the protein level, which are typical targets in the Smad/TGF-β pathway. GED reduced TGF-β-induced fibrotic activation not only in fibroblasts but also in intestinal epithelial cells [105]. A phase II multicentric RCT (SEGMENT) in ulcerative colitis patients was terminated by the sponsor due to recruitment problems [106].

There are several clinical trials investigating the effect of PPARγ agonists on the progression of IBD and fibrosis.

MBF-118, a PPARγ agonist, has successfully completed a phase I trial in healthy volunteers, and a phase IIa trial in Crohn’s disease is underway [107].

In conclusion, PPARγ agonists are potential therapeutic alternatives based on anti-inflammatory and antifibrotic properties, but further studies are needed to investigate their value in both UC and fibrotic CD.

##### PPARγ and Microscopic Colitis

As mentioned above, microscopic colitis is a non-destructive form of IBD. The Th1/Th17 pathway was increased in the colonic mucosa of both types (LC and CC) of MC [108]. CTGF expression in subepithelial zones was increased in collagenous colitis [109]. PPARγ inhibited TGF-β-induced CTGF expression in aortic smooth muscle cells [110], but the role of PPARγ was not investigated in MC. Eosinophilic granulocytes expressed significantly more TGF-β in CC compared to healthy controls [111]. The composition of subepithelial collagen was altered, deposition of collagen types I, III, and VI was increased, and the imbalance between matrix metalloproteinase (MMP) and its inhibitors (TIMPs) led to abnormal collagen deposition and fibrosis in CC, but the thickness of collagen deposition was not correlated with disease activity [16]. Inflammation promotes an imbalance in the rate of pro- and antifibrotic molecules, e.g., PPARγ, leading to fibrosis. There have been no studies on the role of PPARγ in the pathophysiology of MC, but based on the knowledge of MC and the fibrotic process, PPARγ is a potential therapeutic target for this disease and merits further investigation.

##### PPARγ and Celiac Disease

In a gluten-exclusion animal model, C57BL/6 mice were fed a high-fat diet containing 4.5% gluten or no gluten. In the case of the gluten-free diet, decreased weight gain and adiposity were observed without changes in food intake or lipid excretion, and improved insulin resistance and increased PPARα and -γ expression were detected [112].

In an in vitro model, gliadin p31-43, but not the pα-2 or pα-9 peptides, was delivered to lysosomes and increased oxidative burst; it led to an increase in transgulatminase-2 protein levels by inhibiting ubiquitination. This process induced PPARγ degradation and increased inflammation [113].

In a study of childhood celiac disease, duodenal biopsies were performed in 19 children (6 boys, 13 girls; median age 8 years, range 2–15 years) with newly diagnosed CD, 6 children (3 boys, 3 girls; median age 10 years, range 5–16 years) maintained on a GFD, and 10 controls (7 boys, 3 girls; median age 5.25 years, range 0.33–14 years). PPARγ mRNA expression was significantly reduced in newly diagnosed CeD but normalized after a gluten-free diet. At the protein level, the same result was observed by Western blotting. PPARγ was expressed on CD3+ immune cells with newly diagnosed CeD, but in the gluten-free diet and in healthy controls, it was expressed in other cells, such as enterocytes [114].

These data suggest that PPARγ plays a role in the inflammation of celiac disease and that a gluten-free diet may affect its expression and function. However, it is not clear whether PPARγ is involved in the development of another gluten-related disorder, non-celiac gluten sensitivity (NCGS). Further investigation is necessary to understand its role in NCGS.

##### PPARγ and Eosinophilic Gastrointestinal Disorders (EGIDs)

As mentioned above, PPARγ plays a role in the regulation of fibrosis and wound healing. The result of Th2-mediated imbalance is fibrotic stricture formation in the affected location, which is irreversible.

A translational study investigated the effect of rosiglitazone on fibrosis in eosinophilic esophagitis. In isolated fibroblasts and biopsies from the esophagi of patients with eosinophilic esophagitis (EoE), PPARγ expression was higher in active EoE than in inactive EoE and healthy controls. PPARγ expression may be upregulated by increased IL-4. TGF-β production is significantly decreased by the TZDs rosiglitazone and pioglitazone via inhibition of p38 phosphorylation in a dose-dependent manner. Rosiglitazone significantly decreased collagen-1α1 expression, and this effect was more pronounced than that of budesonide treatment. There are no synergistic effects between rosiglitazone and budesonide [115]. TZDs may be candidates for alternative therapy in EGID-associated fibrosis, but clinical trials are needed to investigate this effect.

##### PPARγ and Allergies in the Gastrointestinal System

PPARγ plays a role in allergic inflammation and in the imbalance of the Th2-mediated immune response. Production of IL-4, IL-5, IL-9, and IL-13 induces eosinophils and goblet cells, switches IgE production by B cells, and activates mast cells. Mast cell activation leads to inflammation and cell death.

In an in vitro study, PPARγ-specific silencing using siRNA in bone marrow-derived mast cells significantly increased IgE-mediated mast cell degranulation. In addition, TNF-α, IL-13 secretion, and COX-2 expression in bone-marrow-derived mast cells were significantly increased by PPARγ silencing. In conclusion, PPARγ is a negative regulator of IgE-mediated mast cell degranulation, activation, and inflammatory response [116].

PPARγ plays a key role in Th2-mediated responses. In an animal study, PPARγ promoted IL-4 production, as well as IL-33 receptor and ST2 expression, in selective T-cell-KO mice. ST2 is a marker of Th2 cells in mice. PPARγ is a versatile regulator; it regulates allergic inflammation and enhances protective Th2 immunity, particularly in the case of infection with the nematode *Heligmosomoides polygyrus* [117].

PPARγ plays a role not only in Th2 differentiation but also in the regulation of type-2 innate lymphoid cells (ILC2s). ILC2s play a key role in the regulation of Th2-mediated inflammation and are involved in helminth infections and allergic reactions. IL-33 treatment induced IL-5 and IL-13 production by ILC2s. The secretion of these cytokines was inhibited by the PPARγ antagonist GW9662 and in PPARγ-knockout mice. PPARγ is a critical regulator not only of ILC2 cytokine secretion but also of eicosanoid production and lipid uptake to regulate CD36 expression [118].

Atopic diseases share common pathomechanism not only in the gastrointestinal system but also in the respiratory system and skin. PPARγ plays a role in regulating the Th2 imbalance and its mediated inflammation, and although a trial of pioglitazone in mild asthma failed [119], dietary modulation of PPARγ could improve or, alternatively, trigger inflammation in allergic reactions in the gastrointestinal system.

#### 2.3.3. Dietary Ligands and Modulators of PPARγ

There are some dietary ligands that act indirectly or as full or partial agonists with varying binding affinity for PPARγ to modulate inflammation in the gut (Table 2).

Quercetin, a well-known antioxidant, acts as an agonist of PPARγ to inhibit the NF-κB pathway and prevent myocardial reperfusion injury [120,131]. Quercetin supplementation for 6 weeks in mice fed a high-fat (HFD) diet improved insulin resistance, reduced liver steatosis, decreased the Firmicutes/Bacteroidetes ratio, and modulated gut microbiota-derived SCFA composition [122]. Based on these results, some studies have investigated quercetin’s bioavailability for the treatment of IBD. Quercetin-loaded microcapsules decreased macroscopic edema and neutrophil activation, attenuated histological involvement, and increased IL-10 production in an acetic acid-induced colitis model in mice [135]. In another study, quercetin and dietary olive oil supplemented with fish oil exerted a synergistic effect to improve intestinal inflammation in rats with DSS-induced colitis [136]. Quercetin is degraded by the gut microbiota, and the resulting products—such as 3,4-dihydroxy phenylacetic acid (3,4-DHPAA) and protocatechuic acid (PCA)—are also potent therapeutic agents to decrease oxidative stress-mediated injury and COX-2 expression in IBD [137,138].

Another well-known antioxidant, resveratrol, had controversial effects on PPARs [139,140,141]. In this study, it was demonstrated that resveratrol is a dual PPARα/γ antagonist [142]. The difference may be due to the different methods and concentrations; in any case, it modulates the PPAR-gamma pathway.

Resveratrol treatment inhibited receptor of advanced glycosylation end products (RAGE) and cholesterol accumulation in THP-1-derived macrophages via activation of PPARγ [143].

Resveratrol treatment improved DSS-induced chronic colitis in mice, decreasing disease activity, rectal bleeding, and mortality. In the resveratrol feeding group, reduced secretion of pro-inflammatory cytokines and expression of COX-2, iNOS, and prostaglandin-E synthase-1 were observed at the protein level [144] In DSS-induced colitis in rats, low-dose resveratrol treatment modulated the gut microbiota composition, increased the rates of *Lactobacilli* and *Bifidobacteria*, and improved colitis. In addition to these investigations, there have been several animal studies on the beneficial effects of resveratrol in experimental colitis [145]. There was a 6-week RCT investigating the effects of 500 mg resveratrol capsule treatment on the course of mild–moderate UC. Resveratrol treatment significantly reduced the levels of CRP, TNF-α, and the activity of NF-κB in isolated peripheral blood mononuclear cells (PBMCs). Moreover, the inflammatory bowel disease questionnaire-9 (IBDQ-9) and colitis activity index scores significantly improved in the resveratrol treatment group [146]. Based on this evidence, resveratrol may be a beneficial supplement for improving IBD.

Moreover, the effects of resveratrol were investigated in Th2-mediated intestinal diseases. An in vitro study showed that trans-resveratrol from red wine inhibits eosinophil degranulation and activation at 100 μM concentrations without inducing apoptosis [147]. In an animal model of food allergy, mice were fed with a placebo or resveratrol. After sensitization with intragastric ovalbumin administration and mucosal adjuvant cholera toxin, anaphylaxis, T-cell activation, and ovalbumin-specific IgE, IL-4, IL-13, and IFN-α production were significantly reduced in the resveratrol group compared to the placebo group, thus, resveratrol supplementation may be effective in the prevention of food allergies [148]. In another murine model study on eosinophilic esophagitis, resveratrol treatment significantly reduced the level of miR-223 and improved the eosinophil-related inflammation (esophageal enlargement and eosinophilic infiltration) in *Aspergillus fumigatus*-induced EoE. The miR-223 level was significantly elevated in biopsy specimens of EoE patients, which was correlated with the disease [149]. MiR-223 is a crucial mediator of PPARγ-related M2 activation [150] and regulates eosinophilic degranulation in allergic rhinitis [151]. Resveratrol may have a beneficial effect on modulating Th2-mediated intestinal diseases.

Eucalyptol (1,8-cineol) is a monoterpenoid oxide with antioxidant activity; its administration reduced the activation of colonic inflammation, decreased MPO activity, protected the integrity of surface epithelial structure, and reduced crypt aberration in histological samples from mice with DSS-induced colitis, in a PPARγ-dependent manner [125].

Rosmarinic acid is a polyphenol with a binding affinity for PPARγ [152], but it exhibits no transactivation activity, only modulating the receptor or exerting an indirect effect. Rosmarinic acid significantly decreased inflammatory cytokines, improved DSS-induced colitis and modulated the gut microbiome [127]. In another study, rosmarinic acid modulated bile acid metabolism decreased inflammasome activation, and modulated mucus secretion in a murine colitis model [153]. In a separate investigation, rosmarinic acid-coated nanovesicles were shown to improve DSS-induced colitis in mice. Both free rosmarinic acid and rosmarinic acid-coated vesicles reduced MPO activity and TNF-α levels; however, rosmarinic acid-coated vesicles also decreased NLRP3 inflammasome activity, while free rosmarinic acid did not. This formulation could increase rosmarinic acid’s effectivity and bioavailability [154]. There are not yet any clinical studies on its impact on the clinical course of IBD.

Curcumin has potent anti-inflammatory activity, but its PPARγ agonism is controversial. There are studies confirming that curcumin is a PPARγ ligand. In an in vitro experiment, ethyl-alcohol extraction of curcumin showed PPARγ ligand-binding activity in human preadipocytes, and this affinity was more potent than that of the PPARγ ligand troglitazone [155]. In another study, PPARγ was upregulated by curcumin treatment in hepatic stellate cells in vitro; moreover, curcumin treatment inhibited the proliferation and activation of these cells, and it significantly inhibited the expression of α-SMA and collagen-1; these effects were mediated by PPARγ [156]. In a contrasting study, after curcumin treatment, curcumin was inactive during reporter luciferase assay, in contrast to ciglitazone treatment, and did not bind to the ligand-binding domain of PPARγ. Curcumin did not bind to or activate PPARγ in a competitive ligand-binding assay; rather, it may have indirectly affected PPARγ signaling or modulated another concomitant pathway. However, the formulation of curcumin was not standardized, which may have led to these contrasting results [157]. Curcumin treatment effectively suppressed angiotensin-II-mediated inflammation in rat vascular smooth muscle cells in vitro. PPARγ was translocated and bound to PPREs in a concentration-dependent manner after curcumin pretreatment, and this process was inhibited after treatment with the PPARγ antagonist GW9662, but this inhibitory effect was not complete. This suggests that curcumin exerts anti-inflammatory action in another PPARγ-independent manner [158]. However, the exact effect is not entirely clear even today.

Curcumin significantly decreased IL-12 production by LPS-stimulated macrophages and INF-γ production by CD4+ T cells to suppress Th1 activation in vitro [159]. In an animal model, 0.25% curcumin-containing chow pretreatment for 5 days induced colitis with dinitrobenzenesulfonic acid (DNB) in mice. Curcumin decreased macroscopic inflammation, improved mucosal ulceration, thickened the wall, and promoted significant infiltration with inflammatory cells and myeloperoxidase activity in histological samples [160].

There have been some clinical studies on the effects of curcumin on inflammatory bowel diseases, including a few examples of RCTs with curcumin in IBD.

In a pilot study involving five patients with ulcerative proctitis and five with Crohn’s disease, curcumin improved inflammation, reduced laboratory parameters (CRP, We), and reduced medication and the clinical disease activity index (CDAI) [161].

In a multicenter, placebo-controlled, double-blind trial, 50 mesalamine-treated patients with active mild–moderate UC who failed to respond to an additional 2 weeks of maximum-dose oral and topical mesalamine therapy were enrolled; 26 patients took 3 g/day of curcumin capsules or identical placebo (*n* = 24) for 1 month with mesalamine. Curcumin+5-ASA therapy was more effective than the combination of placebo and mesalamine in inducing clinical and endoscopic remission without adverse events [162]. In another randomized, double-blind, controlled trial (*n* = 56), 80 mg curcumin nanomicelles were taken orally three times per day in combination with mesalamine (3 g/day). The curcuminoids coated with nanomicelles had better bioavailability. Curcumin treatment was significantly associated with improved self-reported well-being, reduced clinical activity of UC, and improved quality of life [163]. Based on these clinical trials, curcumin shows promise as an effective complementary treatment for IBD.

Apigenin modulates PPARγ [132] and is a bioflavonoid with a phenolic structure. This lipophilic molecule is insoluble in water and inactivated in the acidic environment of the stomach, resulting in poor bioavailability. Apigenin treatment inhibited colon damage, MPO activity, and the production of IL-1β, TNF-α, and IL-6 in DSS-induced colitis, as well as the NF-κB and STAT3 pathways in colitis-associated colon cancer tissues in mice [164]. In a different animal study, apigenin treatment improved DSS-induced colitis in mice, and this improvement was comparable to that provided by 5-ASA treatment. Apigenin treatment increased the protein and mRNA expression of claudin-1, as well as the mRNA expression of occludin and claudin-3, which led to decreasing intestinal permeability. PPARγ expression was increased after treatment with 50 mg/kg apigenin, and it affected the MAPK signaling pathway and decreased the *Bacteroides*/ *Desulfovibrio* ratio. The authors suggested that these effects could be considered to be at least partially involved in PPARγ signaling [165]. In another animal study, apigenin increased mucin secretion and goblet cell numbers, decreased pro-inflammatory cytokine secretion, and modulated gut microbiome composition, leading to reduced colonic injury in mice with DSS-induced colitis. There have been no clinical trials, but this formulation could increase bioavailability, and further studies are needed to investigate this promising molecule [130,166]. Experimental studies have been conducted on the formulation of apigenin. In an animal study, treatment with apigenin-Mn(II)-loaded hyaluronic acid nanoparticles significantly improved colitis, significantly decreased MPO activity, inflammatory cytokine secretion, and oxidative injury, and had no side effects in mice with DSS-induced colitis. These effects were comparable to those of the 5-ASA treatment. This formulation is promising for clinical trials in UC [167].

Glutamine is an important L-α-amino acid that is involved in various metabolic processes that help regulate the balance of the immune system. Leukocytes use glutamine, as well as glucose, as an energy substrate for leukocytes. Glutamine is involved in intracellular pathways associated with cell proliferation, tissue repair, apoptosis, and pathogen recognition. There have been several studies on the role of PPARγ in glutamine metabolism and the role of glutamine in regulating PPARγ expression. Glutamine metabolism plays a key role in the regulation of alternative activation of macrophages, and PPARγ is a checkpoint in this process [168]. Glutamine catabolizes into α-ketoglutarate, which enters the tricarboxylic acid cycle. Glutamine, through α-ketoglutarate production, promotes M2 activation, suppresses M1 activation, and is responsible for metabolic reprogramming during M2 activation [169]. However, glutamine not only plays a role in the regulation of M2 activation but also promotes the antitumor activity of tumor-infiltrating conventional dendritic cells (cDCs) [170].

The effect of glutamine supplementation on immune-mediated bowel disease is controversial.

Glutamine has a protective effect on enterocytes and activates PPARγ in a dose- and time-dependent manner, but it does not bind directly. The activation process is not direct, taking place via increased production of the endogenous ligands—15-S-hydroxyeicosatetraenoic acid (15-S-HETE) and dehydrogenated 13-hydroxyoctaolecadienoic acid (13-OXO-ODE) [134].

In a murine study, L-glutamine supplementation improved DSS-induced colitis, decreased macroscopic damage, decreased TNF-α production in parallel, improved the disease activity index, and induced MKP-1, which inhibited cytosolic phospholipase A2 (cPLA2) [171]. In a different animal study, glutamine supplementation inhibited NF-κB and STAT signaling, which led to decreased inflammatory cytokine secretion and less oxidative injury in TNBS-induced colitis [172].

There have been some clinical trials investigating the effects of glutamine supplementation in IBD. In one systematic review, seven studies on glutamine supplementation were evaluated; although there were some studies in which glutamine improved intestinal permeability [173,174], in conclusion, glutamine supplementation had no significant effect on intestinal permeability, disease progression, oxidative stress-induced injury, or inflammatory parameters [175].

One Cochrane analysis investigated the role of glutamine in inducing the remission of Crohn’s disease. The study considered only two small RCTs, and based on its author’s conclusions, further evidence is needed to determine the safety and benefit of glutamine supplementation in active Crohn’s disease [176]. Indeed, there is limited data about the effects of glutamine supplementation in IBD and other immune-mediated gut diseases.

Fermented soy milk (FSM4) is rich in flavonoids, enriched in L. plantarum NCU001563 + S. thermophi-lusNCU074001, and has reduced levels of anti-nutrients (e.g., trypsin inhibitors, phytic acid). FSM4 treatment improved DSS-induced colitis, reduced macroscopic and microscopic damage, and reduced weight loss in mice, as well as inhibiting the expression of TNF-α, IL-6, IL-β, and COX-2 and promoting PPARγ expression; in addition, FSM4 modulated the gut microbiota composition and SCFA contents to regulate PPARγ [177].

In conclusion, several promising molecules have been isolated from phytochemicals and food sources. Some dietary ligands and modulators of PPARγ take advantage of the anti-inflammatory properties of this pathway. Naturally, modulations on other pathways could also play a role in the effects of these phytochemicals that are candidates for complementary therapy in IBD; quercetin, resveratrol, and curcumin seem to be the most promising molecules. Glutamine plays an important role in the regulation of immune responses and immunometabolism, but more evidence is needed to determine whether glutamine supplementation has a place in complementary treatment. Functional foods such as fermented soy milk may modulate the severity of ulcerative colitis, but more research is needed to confirm this effect.

### 2.4. PPARβ/δ

#### 2.4.1. PPARδ’s Structure and Functions 

The third PPAR isoform is PPARδ.

PPARδ is ubiquitously expressed in skin, adipose tissue, enterocytes, muscles, the heart, immune cells, and various types of cancer.

PPARδ is highly conserved; it shows 90% homology between humans and rats. The homozygous knockout of the *ppard* gene led to lethality in murine embryos, and PPARδ played a key role in the placenta and embryogenesis [178].

Like other PPARs, PPARδ forms a heterodimer with RXR, and coactivator proteins and ligand binding lead to its function as a transcription factor, regulating the expression of target genes by binding PPREs in their promoters. PPARδ forms a complex with the transcription factor Bcl-6; this interaction inhibits the repression of pro-inflammatory cytokine genes. This leads to a pro-inflammatory effect that is reversed by PPARδ agonists. In addition to the Bcl-6 interaction, the NF-κB pathway is also regulated by PPARδ [179].

The ligand-binding domain has properties similar to those of the three PPAR isoforms. Not only are there specific PPAR agonists, but there are also dual- and pan-PPAR agonists [180].

Natural ligands for PPARδ include polyunsaturated fatty acids and their metabolites.

A selective PPARδ agonist, GW501516, was given to patients (*n* = 268) with high-density lipoprotein (HDL) cholesterol < 1.16 mmol/L for 12 weeks. LDL and triglyceride levels were significantly reduced in a dose-dependent manner, while HDL and insulin sensitivity were significantly increased compared to placebo [181]. This treatment has the potential to exert an anti-atherogenic effect via increased fatty acid β-oxidation and thermogenesis.

Activation of PPARδ by GW501516 led to increased proglucagon expression and glucagon-like peptide 1 (GLP-1) production by intestinal neuroendocrine L cells in vitro and in vivo; this led to an improvement in insulin sensitivity [145].

PPARδ exerts a protective effect against diet-induced obesity. In the case of specific knockout of PPARδ in intestinal epithelial cells, mice had significantly impaired insulin resistance, and their plasma HDL could not increase after GW501516 stimulation. PPARδ is a potential target for the treatment of dyslipidemia [5].

PPARδ was upregulated in the skin in murine models of psoriasis vulgaris and atopic dermatitis, indicating that PPARδ could be a potential therapeutic target in atopic skin. Treatment with GW501516 reduced atopic skin thickness and the infiltration of CD4+ and CD8+ T cells in mice [182]. This model of atopic dermatitis is relevant for other Th2-mediated diseases, such as in the pathogenesis of food allergies; this is a well-known relationship. In atopic skin, there is an impaired skin barrier, which is a route to food sensitization and plays a role in the progression of food allergies [183]. It can be assumed that PPARδ plays a role not only in the pathogenesis of atopic dermatitis but also in the development of food allergies. For this reason, further research is needed on the role of PPARδ in the field of Th2-mediated immune diseases.

#### 2.4.2. PPARδ, Colonic Inflammation, and Colonic Tumorigenesis

PPARδ plays a role in the regulation of macrophage polarization; it directly regulates CD300a expression in macrophages. CD300a is an inhibitory immunoreceptor with ITIM motifs that inhibits the TLR4 pathway. CD300a-KO mice fed a high-fat diet were susceptible to gut inflammation and showed increased CD68 expression from macrophages and enhanced TLR4-mediated IL-6 secretion [184]. CD300a is expressed constitutively not only in macrophages but also in mast cells. Crosslinking of CD300a and immune complexes inhibited the IgE-mediated activation of mast cells. PPARδ may play a role in the regulation of mast cell activation [185].

PPARδ not only plays a role in improving insulin sensitivity and inflammation but also affects the microbiota composition of the gut. Paneth cells in Lieberkühn crypts recognize antigens through TLRs and secrete alpha-defensins and antimicrobial peptides to control the local microbiome. It is known that, in ileal CD, the number of Paneth cells and the secretion of defensins are reduced. PPARδ plays a key role in Paneth cell differentiation via the Hedgehog pathway, and PPARδ-KO mice have significantly reduced Paneth cell numbers. PPARδ regulates Paneth cells’ maturation and contributes to the gut microbiota composition [186]. Paneth cell metaplasia in the distal colon is a possible early sign of IBD [187]. PPARδ may play a role in this process, and IBD patients have increased susceptibility to colorectal cancer.

Despite their improvement of lipid and insulin metabolism, there are no PPARδ agonists in clinical use for this indication due to their controversial role in tumorigenesis. PPARδ upregulation is associated with reduced metastasis-free survival in various types of cancer, e.g., colorectal cancer, where PPARδ plays a role in invasion, angiogenesis, and migration [188].

In the case of intestinal epithelial cells of PPARδ-KO mice, mice were injected intraperitoneally with the carcinogen azoxymethane (AOM) to create an animal model of AOM-induced carcinogenesis in the colon. Against this genetic background, colon tumorigenesis and tumor-associated angiogenesis were decreased in this animal model. PPARδ is involved in tumorigenesis in colorectal cancers [189]. Sequence analysis of *ppard* from 303 primary tumors of 50 patients with two affected first-degree relatives, 50 sporadic patients, and 360 healthy controls, as well as six colon cancer cell lines, 22 variants were detected; the c.489T>C (p.N163N; rs2076167) variant was associated with worse-differentiated colorectal cancer. PPARδ may play a role in the pathogenesis of colorectal cancer’s development [190].

Although the risk of cancer is uncertain, there is one PPARδ agonist that has undergone clinical trials in primary biliary cirrhosis (PBC). ENHANCE was a phase III study of the efficacy and safety of daily 5 mg seladelpar (*n* = 89), 10 mg seladelpar (*n* = 89), and placebo (*n* = 87) (with UDCA). Treatment with 10 mg of seladelpar improved aminotransferases and pruritus without serious adverse events [191]. This molecule is a candidate for the first PPARδ agonist in clinical use.

In conclusion, PPARδ plays a role in the regulation of colonic inflammation, atopic diseases, and colon tumorigenesis.

## 3. Discussion

PPARs are nuclear receptors and function as transcription factors to regulate the expression of target genes. PPARs have a conserved structure, and they form functionally active heterodimers with RXR [27]. Although they share homology, they have different expression patterns, ligands, and target genes. PPARs have similar transcriptional activities: transactivation and ligand-dependent and -independent transrepression. The regulatory mechanisms of these processes may play a role in the pathogenesis of immune-mediated diseases beyond the gastrointestinal system.

Several post-translational modifications influence the structure and functions of the proteins, thereby regulating the transcriptional activity of PPARs [29]; one of these mechanisms is the ligand-induced SUMOylation of the ligand-binding domain of PPARs [192]. In the case of PPARγ, ligand-dependent SUMOylation influences its anti-inflammatory action in LPS-mediated pathways, and the PPARγ agonist rosiglitazone was responsible for this action [192]. TGF-β1/2 treatment induced MAP kinase and subsequent PPARγ phosphorylation, resulting in the repression of PPARγ in the THP-1 macrophage cell line [58]. It should be noted that there are many connections between TGF-β signaling and the MAPK pathway, and treatment with MAP kinase inhibitors has several effects on the expression of TGF-β and its receptor in human dermal fibroblasts [193]. Post-translational modifications influenced the anti-inflammatory activity of PPARγ, and these pathways could be potential therapeutic targets, meriting further research in the field of immune-mediated gut diseases and intestinal fibrosis.

There is a bidirectional relationship between the gut microbiota and PPARs. PPARα -/- KO mice have increased susceptibility to colitis and commensal dysbiosis [39]. There have been some studies on the modulation of the PPARα pathway by bacteria. When *Lactobacillus kefiri DH5* treatment was given to HFD-fed mice, hepatic steatosis was significantly higher in the control group, while PPARα expression was significantly upregulated in the adipose tissue of the *Lactobacillus*-treated group [194]. In another study on DSS-induced colitis in mice, *Lactobacillus paracasei* B21060 was added 7 days before (pretreatment group) or 2 days after (treatment group) the DSS treatment. There was significantly reduced weight loss and colon damage in both groups compared to the DSS-treated group. PPARγ expression levels were significantly upregulated in both groups with *Lactobacillus paracasei* B21060 [195]. Bacterial metabolites such as SCFAs modulate the expression of PPARγ. Propionate, acetate, and butyrate could increase PPARγ expression in intestinal epithelial cells in vitro, but *Prevotella copri* and *Atopobium parvulum* could mediate PPARγ activation via ERK phosphorylation. In addition to organic acids, there are other bacterial compounds that can be used to regulate PPARγ activation [196]. Further evidence exists for the role of microbial-derived organic acids in influencing PPARγ activation. Streptomycin induced significant depletion of butyrate-producing bacteria in the cecum of mice, and the expression of Angptl4—a gene positively regulated by PPARγ—was measured. Angptl14 expression was significantly reduced after streptomycin treatment, while the opposite was true for NOS-2 (nitric oxide synthase). PPARγ negatively regulated NOS-2 expression. After treatment with rosiglitazone, this process was revised. The effect after treatment with the PPARγ antagonist GW9662 was similar to that of streptomycin. Epithelial PPARγ signaling is responsible for luminal nitrate bioavailability. Streptomycin-induced decreases in PPARγ signaling lead to the expansion of *Enterobacteriaceae,* which is a marker of dysbiosis and epithelial dysfunction [197].

In another study, 5-ASA treatment in mice with DSS-induced colitis led to upregulated PPARγ expression in intestinal epithelial cells and reduced dysbiosis by *Escherichia coli* [198]. Modulation of the gut microbiota with drugs (i.e., antibiotics), dietary interventions, functional foods, and food supplements could influence PPARγ signaling. Conversely, the PPARγ agonist 5-ASA could modulate dysbiosis and the gut microflora.

PPARα plays a role in the regulation of mitochondrial and peroxisomal fatty acid oxidation processes [27] and innate immune responses, e.g., modulation of TLR pathways, inhibition of iNOS production, and reduced activation of the NLRP3 inflammasome [33]. There have been controversial animal studies on experimental colitis in different animal models with the PPARα agonist fenofibrate [49,50], and there is an ongoing phase II study on its use in ulcerative colitis, although the results are yet to be reported [51]. Therefore, further investigations of fibrates are necessary.

Atorvastatin, which has pleiotropic effects and PPARα-mediated anti-inflammatory and antifibrotic properties, is currently undergoing clinical trials [53]. In 64 patients with mild–moderate UC, atorvastatin treatment had no beneficial effect, but a paradoxical disease flare was observed in some patients [199].

Another ongoing clinical trial on the colorectal cancer risk associated with the use of atorvastatin in UC patients has yet to yield results [200]. The natural agonist of PPARα, PEA, has anti-inflammatory properties, but there have been no clinical trials on its effects on the course of IBD; however, there is a pilot phase IIb RCT on its use in irritable bowel syndrome (IBS). Although there are some inflammatory and innate immune components in the pathogenesis of IBS, it is defined as a functional disorder of the gastrointestinal system. PEA treatment was effective in improving abdominal pain; further studies are needed on the disease management of IBD [201]. PPARα could be a useful biomarker of clinical response, and its agonists could be candidates for complementary therapy in IBDs that are commonly associated with IBS-like symptoms.

PPARα could play a role in allergic diseases. PPARα was expressed in eosinophilic granulocytes and downregulated in allergic inflammation and eosinophil activation in vitro and in murine models of asthma [202]. In another study, eosinophils were isolated from peripheral blood; eosinophil survival was significantly reduced by treatment with PPAR agonists (GW7647 and docosahexaenoic acid (DHA)), in a dose- and time-dependent manner [203]. Further studies are needed on their effects on the course of IBD, and there have been no clinical trials on their effects on allergic diseases. These agonists could be potential alternative targets in allergic and atopic diseases, not just in Th1/Th17-mediated diseases.

PPARγ is the best-known member of the PPAR family and plays a role in Th1-mediated and Th2-mediated allergic diseases. The TZDs—synthetic PPARγ agonists—are insulin sensitizers. In addition to antidiabetic and anti-inflammatory effects, PPARγ inhibits fibrotic processes. 5-ASA is a widely used drug in IBD and MC, and the anti-inflammatory effect of 5-ASA is mediated by PPARγ, but it does not affect the course of fibrosis. This antifibrotic property is important to resolve the form of fibrotic strictures in IBD, especially in Crohn’s disease, but also in the collagenous type of MC and in Th2-mediated EGIDs. Unfortunately, the RCT on the 5-ASA analog GED was interrupted due to recruitment problems [105,106], but there is an ongoing phase II RCT with a novel partial PPARγ agonist called MBF-118, which has antifibrotic properties and is well tolerated in animal studies [107]. We summarize the results of clinical studies on PPAR agonists in immune-mediated intestinal diseases in Table 3.

PPARγ agonists could represent ideal drug candidates to work specifically at the site of fibrosis in the gastrointestinal tract in cases without absorption, such as 5-ASA. PPARγ and other PPAR agonists have various systemic adverse effects on heart failure [68], tumorigenesis, myalgia, and hepatic events [204], but they can hopefully be used to develop a safe drug for the treatment of fibrotic strictures. There are some food sources that contain dietary ligands and modulators of PPARγ, especially curcumin, and quercetin, which represent candidates for adjunctive therapy to modulate disease progression and remission in both forms of IBD [138,161,162,163]. There have been some studies about the effects of glutamine on the course of IBD. The animal studies were promising, but the clinical trials are controversial and have limiting factors. Further studies are needed on the exact effects of disease course [176]. In conclusion, dietary intervention with functional foods or supplements, e.g., fermented soymilk [177], may modulate the disease course alongside pharmacological therapy.

PPARδ participates in embryogenesis, tumorigenesis, and colonic inflammation; it also plays a pivotal role in Paneth cell differentiation [186]. A recently published study showed that PPARδ plays a key role in metabolic reprogramming during memory T-cell differentiation; PPARδ suppressed anaerobic glycolysis and increased oxidative metabolism and fatty acid oxidation [205]. PPARδ plays a role not only in this process but also in the mesenchymal metabolic reprogramming of mesenchymal stem cells, which influences immunoregulatory functions in Th1/Th17 cells [206].

The role of PPARδ is very similar in tumor cells. PPARδ-mediated metabolic reprogramming in tumor cells led to the inhibition of anaerobic glycolysis and increased oxidative glutamine and lipid metabolism. This metabolic shift could modulate the functions and survival of tumors and infiltrating immune cells [207]. PPARδ plays a pathogenic role in the development of colorectal cancer cells. In an in vitro study, PPARδ activation induced the expression of the self-renewal regulatory factor Nanog, which led to colorectal stem cell expansion. Knockdown of Nanog inhibited this process during treatment with the PPARδ agonist GW501516. In a murine model of colorectal cancer, Nanog expression was upregulated in normal and cancerous intestinal cells after PPARδ agonist treatment, and it promoted liver metastasis, but it did not affect the size of the tumor. Knockdown of PPARδ completely inhibited this effect on metastasis progression. A high-fat diet-induced colorectal stem cells and liver metastasis in a murine model by inducing Nanog via PPARδ [208]. In another study, PPARδ upregulation was associated with reduced metastasis-free survival in various types of cancer [189], e.g., colorectal cancer.

Based on these findings, PPARδ agonists are not currently used clinically. Only seladelpar is used in primary biliary cirrhosis, but clinical trials indicate that seladelpar may be safe in this indication. In the case of PPARδ ligands, their undefined role in tumorigenesis is an important limiting factor that should be investigated in clinical trials for other indications before being used in clinical practice.

## 4. Conclusions

In summary, PPARs play a role not only in the regulation of lipid metabolism, glucose metabolism, and insulin sensitivity but also in inflammatory and immune responses, cell proliferation, tumorigenesis, modulation of gut microbiota, and fibrotic processes [1,4,11,186]. PPARs have been implicated in the pathogenesis of inflammatory bowel diseases, microscopic colitis, celiac disease, and Th2-mediated allergic diseases. PPARα and PPARγ ligands are promising therapeutic tools for immune-mediated intestinal diseases based on their anti-inflammatory and antifibrotic activity. However, PPARδ has an uncertain role in tumorigenesis, which represents an important factor restricting its introduction in clinical practice. There are many opportunities to develop potential medicines, and the impact of understanding these mechanisms could be profound, but further studies are needed.

## Figures and Tables

**Table 1:** Effects of PPARα on innate immune responses.

Immune Responses Modulated by PPARα	Effects
Transrepression of c-Jun, p65, and Sirt1 [36]	Decreased IL-6 and MCP-1
Transactivation of long noncoding RNA Gm15441 [37]	Blocked NLRP3 inflammasome (mice)
Change in the catabolism of lipid mediators [34]	Decreased inflammatory responses (migration, extravasation, diapedesis)
Inducible nitric oxide synthase (iNOS) 2 downregulation [38]	Inhibition of nitric oxide production; inhibition of effector function of macrophages
Pattern recognition receptor (PRR) (e.g., TLR) modulation [39]	Increased pro-inflammatory cytokine secretion of PPARα -/- KO mice; blocked IL-10 and IL-22 production

**Table 2:** Dietary ligands and modulators of PPARγ.

Ligand	Foods	Effects
Quercetin	Dill, bay leaves, oregano, tarragon, parsley, pomegranate fruit, apples	Inhibits NF-κB [120]; improves cholesterol efflux from macrophages [121]; modulates microbiota ratio in the gut during a high-fat diet in mice [122]
Resveratrol	European blueberries, peanuts, grapes, wine	Regulates cholesterol efflux; modulates the gut microbiome on a high-fat diet; improves dysbiosis [123]
Eucalyptol (1,8-cineol)	*Eucalyptus globulus*, ginger, turmeric	Inhibits NF-κB; improves TNBS-induced colitis in rats; decreases mRNA levels of IL-8 and CXCL1 chemokines [124,125]
Cinnamic acid	Cinnamon	Partial agonist; prevents adipogenesis [126]
Rosmarinic acid	Rosemary, lavender, thyme, sage, marjoram	Improves colitis; regulates intestinal microflora [127]
Curcumin	Turmeric	Anti-inflammatory and antifibrotic action [128]; analogues improve colitis in animal models [129]
Apigenin	Celery, parsley, marjoram, sage, thyme, artichoke, chamomile	Partial agonist; anti-inflammatory; ameliorates colitis; modulates gut microbiota [130,131,132]
Glutamine	Eggs, beef, fish, dairy products	Not a direct PPARγ ligand; stimulates endogenous ligand production; gut-protective effect [133,134]

Abbreviations: NF-κB: nuclear factor kappa-light-chain-enhancer of activated B cells; TNBS: 2,4,6-trinitrobenzenesulfonic acid; CXCL1: chemokine (C-X-C motif) ligand 1.

**Table 3:** Clinical studies on PPAR agonists in the course of immune-mediated gut diseases.

Receptor/Ligand	Disease/Phase	Result
PPARα		
Fibrates	Ulcerative colitis/phase II-III	In progress, awaiting results [51,52]
Atorvastatin	Ulcerative colitis/phase II Ulcerative colitis and colorectal cancer risk/phase II	No beneficial effect/paradoxical flare-up [199] In progress, awaiting results [200]
Palmitoylethanolamide (PEA)	Only animal studies on IBD; no RCTs Irritable bowel syndrome/phase IIb	Improving abdominal pain in IBS patients [201]
PPARγ		
GED-0507-34-Levo (GED)	Ulcerative colitis/phase II	Interrupted due to recruitment problems [106]
MBF-118	Crohn’s disease/phase I–II	In progress, awaiting results [107]
Oral rosiglitazone Rosiglitazone enemas	Mild–moderate ulcerative colitis/phase II Mild–moderate ulcerative colitis/phase II	Clinical improvement and improvement of quality of life [99] Rosiglitazone enemas were effective on distal UC [100]
Oral rosiglitazone	Eosinophilic esophagitis (EoE)/translational research	Improvement of ECM synthesis; RCT needed [115]
